# Supplementary material for: Inequalities in cancer mortality between people with and without disability: A nationwide data linkage study of 10 million adults in Australia
Source: PLoS Med. 2026 Jan 5;23(1):e1004873. doi: 10.1371/journal.pmed.1004873 (PMC12768262; doi:10.1371/journal.pmed.1004873)
Supplement: S1 Methods — (DOCX) [file pmed.1004873.s001.docx]

Supplementary Method

Administrative datasets used to construct the population linkage spine

The spine is based on the combined population from three core datasets: 1) Medicare Consumer Directory (MCD), held by Services Australia, 2) DOMINO Centrelink Administrative Data (DOMINO CAD), held by Australian Department of Social Services, and Personal Income Tax (PIT), held by Australian Taxation Office.

These core datasets were used when constructing the population spine. Majority of the core datasets have been linked using deterministic methodology, whereas some linkages of the core datasets have been performed using probabilistic techniques. Annual spine updates were conducted using most recent data to ensure high quality of the spine.

Disability questions in Australia’s 2011 Census of Population and Housing

Four questions were asked to identify people with disability in the Census. Three questions asked whether the person ever need someone to help with, or be with them for each of the three core activity areas, and one question asked reasons for the need for assistance or supervision. Australian Bureau of Statistics (ABS) releases a summary variable of disability for research purpose. Respondents were categorised as having disability if they had need for assistance in at least one core activity area (self-care, body movement, and communication) and answered long-term health conditions or disability that lasted six months or more as the reason.

Respondents aged 40 years and above who had need for help were also included in this category even if they did not choose long-term condition or disability as the reason. Australian Bureau of Statistics has tested that from age 40, people who need assistance due to a long-term health condition or disability may state that this need is due to ageing. Respondents were categorised as non-disabled if they answered that they had no need for help or supervision with core activities. People were in non-disabled category if their need for assistance was because of short-term health conditions, difficulty with English, young age as the only reason, or reasons other than long-term health conditions and disability. ^1,2^

20. Does the person ever need someone to help with, or be with them for, self-care activities?

For example: doing everyday activities such as eating, showering, dressing or toileting.

- Yes, always.
- Yes, sometimes.
- No

21. Does the person ever need someone to help with, or be with them for, body movement activities? For example: getting out of bed, moving around at home or at places away from home.

- Yes, always.
- Yes, sometimes.
- No

22. Does the person ever need someone to help with, or be with them for, communication activities? For example: understanding, or being understood by, others.

- Yes, always.
- Yes, sometimes.
- No

23. What are the reasons for the need for assistance or supervision shown in questions 20, 21, and 22? Mark all applicable reasons.

- No need for help or supervision
- Short-term health condition (lasting less than six months)
- Long-term health condition (lasting six months or more)
- Disability (lasting six months or more)
- Old or young age
- Difficulty with English language
- Other causes

In Australia, the Survey of Disability, Ageing, and Carers (SDAC) is considered the 'gold standard' for measuring disability. This survey closely aligns with the ICF model and provides a comprehensive assessment of disability. However, the SDAC uses over 160 questions to determine disability status, making it impractical for nation-wide data collection.^3^

The four Census questions on need for assistance with core activities are designed to conceptually align with the subgroup of people with disability with severe or profound limitation identified by the SDAC. The Census questions identify individuals with a profound or severe core activity limitation using similar criteria SDAC uses to identify people needing assistance in their day to day lives in one or more of the three core activity areas of self-care, mobility and communication.^3^ Consequently, the disability group identified by the Census questions does not include people with less severe functional limitations and is not representative of the whole population of people with disability in Australia. The questions do, however, identify individuals with the greatest support needs who are likely to experience the largest inequalities in health and mortality relative to people without disability, and it can be used in large-scale data collection such as in the Census.

Bootstrap confidence intervals

Bootstrapping is a statistical technique that allows us to estimate confidence intervals without relying on strict assumptions about the underlying data distribution. This was done by repeatedly resampling the observed data with replacement to create many simulated datasets. From these, we calculated the inequality estimates across all samples and use the distribution of those results to derive a confidence interval.^4^

**References**

1. Australian Bureau of Statistics (ABS). Census of Population and Housing: Understanding the Census and Census Data, Australia , 2016. 2017. https://www.abs.gov.au/ausstats/abs@.nsf/Lookup/by%20Subject/2900.0~2016~Main%20Features~ASSNP%20Core%20Activity%20Need%20for%20Assistance~10041 (Accessed 19 May 2023).

2. Australian Bureau of Statistics (ABS). 2016 Census Dictionary: Core Activity Need for Assistance. 2016. https://www.abs.gov.au/ausstats/abs@.nsf/lookup/2901.0chapter27102016 (Accessed 19 May 2023).

3. Australian Bureau of Statistics. 2022. Understanding disability statistics in the Census and the Survey of Disability, Ageing and Carers: An explanation of disability data available in the Census and other ABS sources. <https://www.abs.gov.au/statistics/detailed-methodology-information/information-papers/understanding-disability-statistics-census-and-survey-disability-ageing-and-carers>

4. Efron, B., Tibshirani, R. The Bootstrap Method for Assessing Statistical Accuracy. Behaviormetrika 12, 1–35 (1985). https://doi.org/10.2333/bhmk.12.17_1
