# Supplementary material for: Inequalities in cancer mortality between people with and without disability: A nationwide data linkage study of 10 million adults in Australia
Source: PLoS Med. 2026 Jan 5;23(1):e1004873. doi: 10.1371/journal.pmed.1004873 (PMC12768262; doi:10.1371/journal.pmed.1004873)
Supplement: S1 Fig — (DOCX) [file pmed.1004873.s002.docx]

S1 Figure. Age-specific mortality rates due to all cancer and specific cancer types according to disability status, females, age 25 to 74 years, Australia.


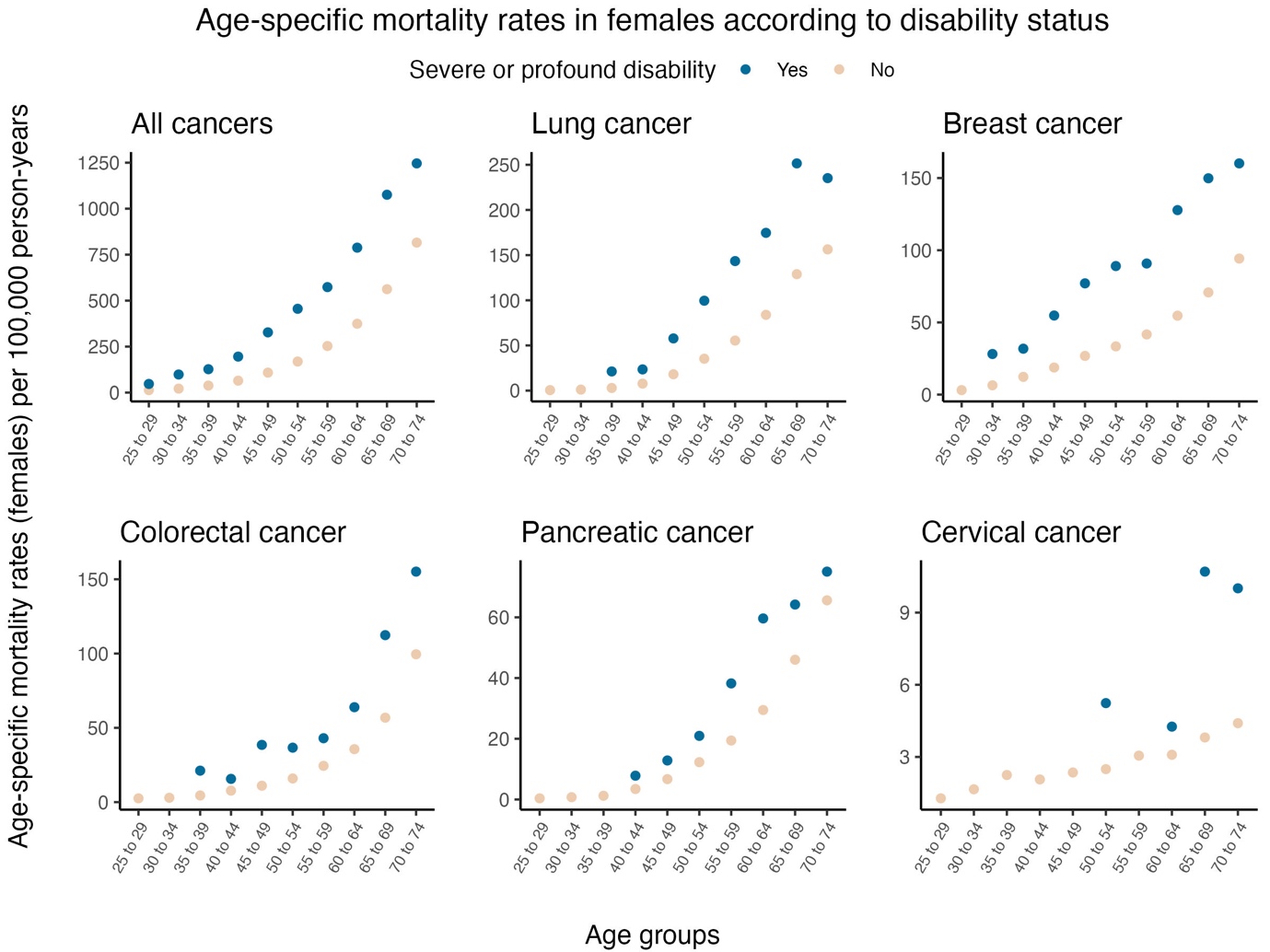


Note: The scale of Y axis varies. Rates based on death counts lower than 10 were suppressed due to confidentiality requirement.
