# Supplementary material for: Inequalities in cancer mortality between people with and without disability: A nationwide data linkage study of 10 million adults in Australia
Source: PLoS Med. 2026 Jan 5;23(1):e1004873. doi: 10.1371/journal.pmed.1004873 (PMC12768262; doi:10.1371/journal.pmed.1004873)
Supplement: S1 Table — (DOCX) [file pmed.1004873.s004.docx]

S1 Table. Lifestyle-related cancers based on assessments of the International Agency for Research on Cancer and the World Cancer Research Fund

| **Groups based on prevention strategies** | **Cancers and ICD-10 codes** |
| --- | --- |
| 1. Smoking-related ^1^ | - Lung (C33–C34), - Oral cavity, and pharynx (C00-C14), - Nasal cavity (C30.0) and accessory sinuses (C31), - Larynx (C32), - Oesophagus (C15), - Stomach (C16), - Pancreas (C25), - Colorectum (C18–C20, C26.0) - Liver (C22), - Kidney (body and pelvis) (C64 and C65), - Ureter (C66), - Urinary bladder (C67), - Uterine cervix and ovary (mucinous) (C53 cervix uteri, C56 Ovary – no histological data to determine whether it is mucinous), - Myeloid leukaemia (C92) |
| 1. Obesity-related ^2^ | - Oesophagus (C15), - Postmenopausal breast (C50, no data to determine pre/post-menopausal), - Liver (C22), - Gallbladder (C23), - Kidney (C64), - Colorectal (C18–C20, C26.0), - Multiple myeloma (C90.0), - Meningioma (C70), - Thyroid (C73), - Gastric cardia (stomach, C16.0), - Pancreatic (C25), - Ovary (C56), - Corpus uteri (uterus) (C54). |
| 1. Alcohol-related ^1^ | - Oral cavity and pharynx (C00-C14), - Larynx (C32), - Oesophagus (C15), - Colorectum (C18–C20, C26.0), - Liver (hepatocellular carcinoma) (C22), - Female breast (C50) - Stomach (C16) |

**References**

1. IARC Working Group on the Evaluation of Carcinogenic Risks to Humans. A review of human carcinogens: personal habits and indoor combustions. IARC Monogr Eval Carcinog Risks Hum 2012; 100: 1.

2. Lauby-Secretan B, Scoccianti C, Loomis D, Grosse Y, Bianchini F, Straif K. International Agency for Research on Cancer Handbook Working Group. Body fatness and cancer—Viewpoint of the IARC working group. N Engl J Med 2016; 375(8): 794-8.
