# Supplementary material for: Inequalities in cancer mortality between people with and without disability: A nationwide data linkage study of 10 million adults in Australia
Source: PLoS Med. 2026 Jan 5;23(1):e1004873. doi: 10.1371/journal.pmed.1004873 (PMC12768262; doi:10.1371/journal.pmed.1004873)
Supplement: S2 Table — (DOCX) [file pmed.1004873.s005.docx]

S2 Table. Crude and age-standardised cancer-specific mortality rates, rate differences, and rate ratios comparing people with and without disability, age 25 to 74 years, Australia.

| Cause of death | People with disability | | | People without disability | | | Rate difference, per 100,000 years | Rate ratio ^b^ |
| --- | --- | --- | --- | --- | --- | --- | --- | --- |
|  | Deaths | Mortality rate, per 100,000 person years | | Deaths | Mortality rate, per 100,000 person years | |  |  |
|  |  | Crude | Age-standardised (95% CI) ^a^ |  | Crude | Age-standardised (95% CI) ^a^ |  |  |
| Females |  |  |  |  |  |  |  |  |
| Lung cancer | 1964 | 128 | 135 (128, 141) | 17225 | 36 | 68 (67, 69) | 67 (60, 73) | 1.98 (1.89, 2.08) |
| Breast cancer | 1497 | 98 | 101 (96, 106) | 14112 | 30 | 47 (46, 47) | 54 (49, 60) | 2.17 (2.05, 2.29) |
| Colorectal cancer | 941 | 61 | 65 (61, 69) | 9111 | 19 | 35 (34, 36) | 30 (25, 34) | 1.85 (1.72, 1.97) |
| Pancreatic cancer | 565 | 37 | 39 (36, 42) | 6394 | 13 | 26 (25, 26) | 13 (10, 17) | 1.53 (1.39, 1.66) |
| Cervical cancer | 87 | 6 | 6 (5, 7) | 1150 | 2 | 3 (3, 3) | 3 (2, 4) | 1.96 (1.53, 2.39) |
| Males |  |  |  |  |  |  |  |  |
| Lung cancer | 2786 | 189 | 204 (197, 212) | 22558 | 52 | 101 (100, 103) | 103 (95, 111) | 2.02 (1.94, 2.10) |
| Colorectal cancer | 1301 | 88 | 97 (91, 102) | 12304 | 28 | 53 (52, 54) | 44 (38, 49) | 1.83 (1.72, 1.94) |
| Prostate cancer | 683 | 46 | 84 (79, 90) | 7783 | 18 | 54 (53, 55) | 31 (26, 36) | 1.57 (1.48, 1.67) |
| Pancreatic cancer | 314 | 21 | 50 (46, 54) | 4365 | 10 | 33 (32, 34) | 17 (13, 20) | 1.50 (1.38, 1.61) |

Abbreviations: CI, confidence interval; RR, risk ratio; RD, risk difference. ^a^ The Standard Population used is people (both males and females) with disability. ^b^ Reference groups for RR and RD are males and females without disability.
