# Supplementary material for: Inequalities in cancer mortality between people with and without disability: A nationwide data linkage study of 10 million adults in Australia
Source: PLoS Med. 2026 Jan 5;23(1):e1004873. doi: 10.1371/journal.pmed.1004873 (PMC12768262; doi:10.1371/journal.pmed.1004873)
Supplement: S3 Table — (DOCX) [file pmed.1004873.s006.docx]

S3 Table. Age-specific overall cancer and type-specific mortality rates, rate differences and rate ratios comparing people with and without disability, age 25 to 74 years, Australia.

Rates and rate differences in per 100,000 person years. Cells based on counts lower than 10 were suppressed as a confidentiality requirement.

| Outcome | Age Group | Female | | | | | | | Male | | | | |
| --- | --- | --- | --- | --- | --- | --- | --- | --- | --- | --- | --- | --- | --- |
|  |  | Rates according to disability status | | | Rate difference | | Rate ratio | | Rates according to disability status | | | Rate difference | Rate ratio |
|  |  | Yes | No |  | |  | | Yes | | No |  | |  |
| All cancers | 25 to 29 | 47 | 14 | 34 | | 3.5 | | 41 | | 11 | 30 | | 3.6 |
|  | 30 to 34 | 98 | 22 | 77 | | 4.6 | | 65 | | 17 | 48 | | 3.9 |
|  | 35 to 39 | 127 | 38 | 89 | | 3.3 | | 117 | | 31 | 86 | | 3.7 |
|  | 40 to 44 | 196 | 65 | 131 | | 3 | | 192 | | 59 | 134 | | 3.3 |
|  | 45 to 49 | 328 | 109 | 219 | | 3 | | 361 | | 116 | 245 | | 3.1 |
|  | 50 to 54 | 456 | 169 | 287 | | 2.7 | | 574 | | 210 | 364 | | 2.7 |
|  | 55 to 59 | 574 | 253 | 320 | | 2.3 | | 777 | | 354 | 423 | | 2.2 |
|  | 60 to 64 | 788 | 374 | 414 | | 2.1 | | 1,066 | | 554 | 512 | | 1.9 |
|  | 65 to 69 | 1,076 | 562 | 514 | | 1.9 | | 1,482 | | 868 | 614 | | 1.7 |
|  | 70 to 74 | 1,246 | 816 | 431 | | 1.5 | | 2,063 | | 1,346 | 717 | | 1.5 |
| Lung cancer | 25 to 29 | . | 1 | . | | . | | . | | 1 | . | | . |
|  | 30 to 34 | . | 1 | . | | . | | . | | 1 | . | | . |
|  | 35 to 39 | 21 | 3 | 18 | | 7.2 | | 11 | | 4 | 7 | | 2.7 |
|  | 40 to 44 | 23 | 8 | 16 | | 3 | | 33 | | 9 | 25 | | 3.8 |
|  | 45 to 49 | 58 | 18 | 40 | | 3.2 | | 92 | | 21 | 71 | | 4.4 |
|  | 50 to 54 | 100 | 35 | 64 | | 2.8 | | 140 | | 43 | 97 | | 3.3 |
|  | 55 to 59 | 143 | 55 | 88 | | 2.6 | | 176 | | 74 | 101 | | 2.4 |
|  | 60 to 64 | 175 | 84 | 91 | | 2.1 | | 256 | | 116 | 139 | | 2.2 |
|  | 65 to 69 | 252 | 129 | 123 | | 2 | | 352 | | 193 | 159 | | 1.8 |
|  | 70 to 74 | 235 | 156 | 79 | | 1.5 | | 440 | | 263 | 176 | | 1.7 |
| Breast cancer | 25 to 29 | . | 3 | . | | . | |  | |  |  | |  |
|  | 30 to 34 | 28 | 6 | 22 | | 4.4 | |  | |  |  | |  |
|  | 35 to 39 | 32 | 12 | 20 | | 2.6 | |  | |  |  | |  |
|  | 40 to 44 | 55 | 19 | 36 | | 2.9 | |  | |  |  | |  |
|  | 45 to 49 | 77 | 27 | 50 | | 2.9 | |  | |  |  | |  |
|  | 50 to 54 | 89 | 33 | 56 | | 2.7 | |  | |  |  | |  |
|  | 55 to 59 | 91 | 42 | 49 | | 2.2 | |  | |  |  | |  |
|  | 60 to 64 | 128 | 55 | 73 | | 2.3 | |  | |  |  | |  |
|  | 65 to 69 | 150 | 71 | 79 | | 2.1 | |  | |  |  | |  |
|  | 70 to 74 | 160 | 94 | 66 | | 1.7 | |  | |  |  | |  |
| Colorectal cancer | 25 to 29 | . | 3 | . | | . | | . | | 3 | . | | . |
|  | 30 to 34 | . | 3 | . | | . | | 13 | | 3 | 10 | | 4.6 |
|  | 35 to 39 | 21 | 4 | 17 | | 4.7 | | 21 | | 5 | 16 | | 4.3 |
|  | 40 to 44 | 16 | 8 | 8 | | 2 | | 25 | | 9 | 16 | | 2.7 |
|  | 45 to 49 | 39 | 11 | 27 | | 3.5 | | 35 | | 15 | 20 | | 2.3 |
|  | 50 to 54 | 37 | 16 | 21 | | 2.3 | | 61 | | 24 | 37 | | 2.5 |
|  | 55 to 59 | 43 | 24 | 19 | | 1.8 | | 75 | | 38 | 37 | | 2 |
|  | 60 to 64 | 64 | 36 | 28 | | 1.8 | | 98 | | 60 | 38 | | 1.6 |
|  | 65 to 69 | 112 | 57 | 56 | | 2 | | 147 | | 82 | 65 | | 1.8 |
|  | 70 to 74 | 155 | 100 | 56 | | 1.6 | | 246 | | 148 | 98 | | 1.7 |
| Prostate cancer | 25 to 29 |  |  |  | |  | | . | | . | . | | . |
|  | 30 to 34 |  |  |  | |  | | . | | . | . | | . |
|  | 35 to 39 |  |  |  | |  | | . | | 0 | . | | . |
|  | 40 to 44 |  |  |  | |  | | . | | 1 | . | | . |
|  | 45 to 49 |  |  |  | |  | | . | | 3 | . | | . |
|  | 50 to 54 |  |  |  | |  | | 18 | | 9 | 9 | | 2 |
|  | 55 to 59 |  |  |  | |  | | 48 | | 24 | 24 | | 2 |
|  | 60 to 64 |  |  |  | |  | | 83 | | 51 | 32 | | 1.6 |
|  | 65 to 69 |  |  |  | |  | | 161 | | 99 | 62 | | 1.6 |
|  | 70 to 74 |  |  |  | |  | | 278 | | 188 | 90 | | 1.5 |
| Pancreatic cancer | 25 to 29 | . | 0 | . | | . | | . | | 0 | . | | . |
|  | 30 to 34 | . | 1 | . | | . | | . | | 1 | . | | . |
|  | 35 to 39 | . | 1 | . | | . | | . | | 2 | . | | . |
|  | 40 to 44 | 8 | 3 | 4 | | 2.3 | | 8 | | 5 | 4 | | 1.8 |
|  | 45 to 49 | 13 | 7 | 6 | | 1.9 | | 21 | | 10 | 11 | | 2.2 |
|  | 50 to 54 | 21 | 12 | 9 | | 1.7 | | 31 | | 17 | 14 | | 1.8 |
|  | 55 to 59 | 38 | 19 | 19 | | 2 | | 48 | | 27 | 21 | | 1.8 |
|  | 60 to 64 | 60 | 29 | 30 | | 2 | | 67 | | 40 | 27 | | 1.7 |
|  | 65 to 69 | 64 | 46 | 18 | | 1.4 | | 83 | | 57 | 26 | | 1.5 |
|  | 70 to 74 | 75 | 66 | 9 | | 1.1 | | 103 | | 83 | 20 | | 1.2 |
| Cervical cancer | 25 to 29 | . | 1 | . | | . | |  | |  |  | |  |
|  | 30 to 34 | . | 2 | . | | . | |  | |  |  | |  |
|  | 35 to 39 | . | 2 | . | | . | |  | |  |  | |  |
|  | 40 to 44 | . | 2 | . | | . | |  | |  |  | |  |
|  | 45 to 49 | . | 2 | . | | . | |  | |  |  | |  |
|  | 50 to 54 | 5 | 2 | 3 | | 2.1 | |  | |  |  | |  |
|  | 55 to 59 | . | 3 | . | | . | |  | |  |  | |  |
|  | 60 to 64 | 4 | 3 | 1 | | 1.4 | |  | |  |  | |  |
|  | 65 to 69 | 11 | 4 | 7 | | 2.8 | |  | |  |  | |  |
|  | 70 to 74 | 10 | 4 | 6 | | 2.3 | |  | |  |  | |  |
| Obesity-related cancers | 25 to 29 | . | 7 | . | | . | | 14 | | 4 | 10 | | 3.4 |
|  | 30 to 34 | 42 | 12 | 30 | | 3.5 | | 26 | | 6 | 20 | | 4.6 |
|  | 35 to 39 | 64 | 22 | 41 | | 2.9 | | 43 | | 12 | 30 | | 3.5 |
|  | 40 to 44 | 110 | 38 | 71 | | 2.9 | | 75 | | 24 | 51 | | 3.1 |
|  | 45 to 49 | 173 | 61 | 112 | | 2.8 | | 120 | | 48 | 72 | | 2.5 |
|  | 50 to 54 | 236 | 89 | 147 | | 2.6 | | 201 | | 83 | 119 | | 2.4 |
|  | 55 to 59 | 272 | 132 | 140 | | 2.1 | | 277 | | 132 | 145 | | 2.1 |
|  | 60 to 64 | 379 | 188 | 191 | | 2 | | 354 | | 193 | 161 | | 1.8 |
|  | 65 to 69 | 508 | 274 | 234 | | 1.9 | | 465 | | 266 | 199 | | 1.7 |
|  | 70 to 74 | 616 | 407 | 209 | | 1.5 | | 634 | | 408 | 226 | | 1.6 |
| Alcohol-related cancers | 25 to 29 | . | 7 | . | | . | | 14 | | 4 | 10 | | 3.4 |
|  | 30 to 34 | 42 | 11 | 32 | | 4 | | 26 | | 5 | 21 | | 4.8 |
|  | 35 to 39 | 64 | 20 | 44 | | 3.2 | | 43 | | 10 | 32 | | 4.1 |
|  | 40 to 44 | 94 | 31 | 63 | | 3 | | 67 | | 20 | 47 | | 3.3 |
|  | 45 to 49 | 148 | 46 | 102 | | 3.2 | | 113 | | 41 | 72 | | 2.7 |
|  | 50 to 54 | 173 | 63 | 110 | | 2.7 | | 201 | | 70 | 131 | | 2.9 |
|  | 55 to 59 | 182 | 88 | 94 | | 2.1 | | 250 | | 109 | 141 | | 2.3 |
|  | 60 to 64 | 264 | 121 | 143 | | 2.2 | | 299 | | 153 | 146 | | 2 |
|  | 65 to 69 | 359 | 173 | 186 | | 2.1 | | 382 | | 206 | 175 | | 1.8 |
|  | 70 to 74 | 420 | 265 | 155 | | 1.6 | | 524 | | 318 | 206 | | 1.6 |
| Smoking-related cancers | 25 to 29 | 16 | 7 | 9 | | 2.3 | | 14 | | 6 | 8 | | 2.3 |
|  | 30 to 34 | 42 | 9 | 33 | | 4.5 | | 39 | | 9 | 30 | | 4.5 |
|  | 35 to 39 | 64 | 17 | 47 | | 3.7 | | 74 | | 19 | 55 | | 3.9 |
|  | 40 to 44 | 86 | 31 | 55 | | 2.8 | | 126 | | 38 | 88 | | 3.3 |
|  | 45 to 49 | 167 | 57 | 110 | | 2.9 | | 240 | | 80 | 161 | | 3 |
|  | 50 to 54 | 257 | 95 | 161 | | 2.7 | | 403 | | 143 | 260 | | 2.8 |
|  | 55 to 59 | 339 | 149 | 190 | | 2.3 | | 522 | | 233 | 288 | | 2.2 |
|  | 60 to 64 | 447 | 222 | 225 | | 2 | | 708 | | 351 | 357 | | 2 |
|  | 65 to 69 | 632 | 343 | 288 | | 1.8 | | 920 | | 528 | 392 | | 1.7 |
|  | 70 to 74 | 721 | 491 | 230 | | 1.5 | | 1,248 | | 777 | 472 | | 1.6 |
| Lifestyle-related cancers | 25 to 29 | 16 | 10 | 6 | | 1.6 | | 14 | | 6 | 8 | | 2.3 |
|  | 30 to 34 | 56 | 16 | 40 | | 3.5 | | 39 | | 9 | 30 | | 4.3 |
|  | 35 to 39 | 95 | 30 | 65 | | 3.2 | | 74 | | 20 | 55 | | 3.8 |
|  | 40 to 44 | 149 | 52 | 97 | | 2.9 | | 126 | | 39 | 87 | | 3.2 |
|  | 45 to 49 | 257 | 88 | 169 | | 2.9 | | 255 | | 82 | 173 | | 3.1 |
|  | 50 to 54 | 372 | 135 | 237 | | 2.7 | | 415 | | 148 | 266 | | 2.8 |
|  | 55 to 59 | 454 | 204 | 250 | | 2.2 | | 548 | | 243 | 306 | | 2.3 |
|  | 60 to 64 | 618 | 297 | 320 | | 2.1 | | 735 | | 367 | 368 | | 2 |
|  | 65 to 69 | 846 | 446 | 400 | | 1.9 | | 978 | | 552 | 426 | | 1.8 |
|  | 70 to 74 | 961 | 631 | 330 | | 1.5 | | 1,320 | | 816 | 503 | | 1.6 |

Rates based on death counts lower than 10 were suppressed due to confidentiality requirement to prevent re-identification of individuals.
