# Supplementary material for: Inequalities in cancer mortality between people with and without disability: A nationwide data linkage study of 10 million adults in Australia
Source: PLoS Med. 2026 Jan 5;23(1):e1004873. doi: 10.1371/journal.pmed.1004873 (PMC12768262; doi:10.1371/journal.pmed.1004873)
Supplement: S4 Table — (DOCX) [file pmed.1004873.s007.docx]

S4 Table. Age structure and prevalence of disability by 5-year age group in people who were linked and not linked to the population spine

| Sex | Age groups | Disability status of people **not linked** to the population spine (excluded) | | | | | Disability status in people **linked** to the population spine (included) | | | | | Disability status **if we were to include the group not linked** | | | | |
| --- | --- | --- | --- | --- | --- | --- | --- | --- | --- | --- | --- | --- | --- | --- | --- | --- |
|  |  | Yes | % | No | % | **Prevalence** | Yes | % | No | % | **Prevalence** | Yes | % | No | % | **Prevalence** |
| Females | 25 to 29 | 1515 | 3.9 | 113072 | 13.1 | **1.3** | 7088 | 3.6 | 594113 | 11.3 | **1.2** | 8603 | 3.7 | 707185 | 11.6 | **1.2** |
| Females | 30 to 34 | 1545 | 4.0 | 94789 | 10.9 | **1.6** | 8000 | 4.1 | 590614 | 11.3 | **1.3** | 9545 | 4.1 | 685403 | 11.2 | **1.4** |
| Females | 35 to 39 | 2015 | 5.2 | 93604 | 10.8 | **2.1** | 10730 | 5.5 | 628930 | 12.0 | **1.7** | 12745 | 5.4 | 722534 | 11.8 | **1.7** |
| Females | 40 to 44 | 2895 | 7.5 | 101049 | 11.7 | **2.8** | 14660 | 7.5 | 633743 | 12.1 | **2.3** | 17555 | 7.5 | 734792 | 12.0 | **2.3** |
| Females | 45 to 49 | 3567 | 9.2 | 103619 | 12.0 | **3.3** | 18310 | 9.3 | 604322 | 11.5 | **2.9** | 21877 | 9.3 | 707941 | 11.6 | **3.0** |
| Females | 50 to 54 | 4439 | 11.4 | 103913 | 12.0 | **4.1** | 22873 | 11.7 | 572959 | 10.9 | **3.8** | 27312 | 11.6 | 676872 | 11.1 | **3.9** |
| Females | 55 to 59 | 4961 | 12.8 | 93429 | 10.8 | **5.0** | 25718 | 13.1 | 507477 | 9.7 | **4.8** | 30679 | 13.1 | 600906 | 9.8 | **4.9** |
| Females | 60 to 64 | 5471 | 14.1 | 75271 | 8.7 | **6.8** | 30305 | 15.5 | 471486 | 9.0 | **6.0** | 35776 | 15.2 | 546757 | 9.0 | **6.1** |
| Females | 65 to 69 | 5249 | 13.5 | 50198 | 5.8 | **9.5** | 26560 | 13.6 | 361155 | 6.9 | **6.9** | 31809 | 13.6 | 411353 | 6.7 | **7.2** |
| Females | 70 to 74 | 7120 | 18.4 | 37160 | 4.3 | **16.1** | 31627 | 16.1 | 271149 | 5.2 | **10.4** | 38747 | 16.5 | 308309 | 5.1 | **11.2** |
| Males | 25 to 29 | 2205 | 5.4 | 140555 | 14.6 | **1.5** | 8222 | 4.2 | 542411 | 11.2 | **1.5** | 10427 | 4.4 | 682966 | 11.7 | **1.5** |
| Males | 30 to 34 | 2041 | 5.0 | 115215 | 12.0 | **1.7** | 8696 | 4.4 | 541174 | 11.1 | **1.6** | 10737 | 4.5 | 656389 | 11.3 | **1.6** |
| Males | 35 to 39 | 2469 | 6.1 | 111083 | 11.5 | **2.2** | 10749 | 5.5 | 573288 | 11.8 | **1.8** | 13218 | 5.6 | 684371 | 11.8 | **1.9** |
| Males | 40 to 44 | 3186 | 7.9 | 115543 | 12.0 | **2.7** | 13901 | 7.1 | 574432 | 11.8 | **2.4** | 17087 | 7.2 | 689975 | 11.9 | **2.4** |
| Males | 45 to 49 | 3701 | 9.1 | 115083 | 11.9 | **3.1** | 16844 | 8.6 | 561436 | 11.6 | **2.9** | 20545 | 8.7 | 676519 | 11.6 | **2.9** |
| Males | 50 to 54 | 4452 | 11.0 | 110473 | 11.5 | **3.9** | 20259 | 10.3 | 535799 | 11.0 | **3.6** | 24711 | 10.4 | 646272 | 11.1 | **3.7** |
| Males | 55 to 59 | 4678 | 11.5 | 94156 | 9.8 | **4.7** | 24143 | 12.3 | 480082 | 9.9 | **4.8** | 28821 | 12.2 | 574238 | 9.9 | **4.8** |
| Males | 60 to 64 | 6604 | 16.3 | 78079 | 8.1 | **7.8** | 34113 | 17.4 | 447998 | 9.2 | **7.1** | 40717 | 17.2 | 526077 | 9.0 | **7.2** |
| Males | 65 to 69 | 5778 | 14.2 | 50065 | 5.2 | **10.3** | 30933 | 15.7 | 345028 | 7.1 | **8.2** | 36711 | 15.5 | 395093 | 6.8 | **8.5** |
| Males | 70 to 74 | 5470 | 13.5 | 32884 | 3.4 | **14.3** | 28636 | 14.6 | 256977 | 5.3 | **10.0** | 34106 | 14.4 | 289861 | 5.0 | **10.5** |
