# Supplementary material for: Inequalities in cancer mortality between people with and without disability: A nationwide data linkage study of 10 million adults in Australia
Source: PLoS Med. 2026 Jan 5;23(1):e1004873. doi: 10.1371/journal.pmed.1004873 (PMC12768262; doi:10.1371/journal.pmed.1004873)
Supplement: S5 Table — (DOCX) [file pmed.1004873.s008.docx]

S5 Table. Age-standardised overall cancer mortality rate differences and rate ratios from sensitivity analysis for missing disability status

| Measures of inequality | Point estimate | 95% CI | | Point estimate | 95% CI | | Point estimate | 95% CI | |
| --- | --- | --- | --- | --- | --- | --- | --- | --- | --- |
|  | Main analysis * | | | Categorising missing disability status to “no disability” | | | Categorising missing disability to “disability” | | |
| **Female** |  |  |  |  |  |  |  |  |  |
| Age-standardised rate difference | **314** | 301 | 328 | **313** | 301 | 324 | **272** | 261 | 283 |
| Age-standardised rate ratio | **1.96** | 1.92 | 2.00 | **1.95** | 1.91 | 1.99 | **1.83** | 1.80 | 1.87 |
| **Male** |  |  |  |  |  |  |  |  |  |
| Age-standardised rate difference | **410** | 394 | 427 | **408** | 390 | 425 | **366** | 351 | 380 |
| Age-standardised rate ratio | **1.83** | 1.80 | 1.87 | **1.82** | 1.79 | 1.86 | **1.74** | 1.71 | 1.77 |

Abbreviation: CI – confidence intervals. * Results from the main analysis presented in this table for comparison.
