# Supplementary material for: Inequalities in cancer mortality between people with and without disability: A nationwide data linkage study of 10 million adults in Australia
Source: PLoS Med. 2026 Jan 5;23(1):e1004873. doi: 10.1371/journal.pmed.1004873 (PMC12768262; doi:10.1371/journal.pmed.1004873)
Supplement: S1 Checklist — An Explanation and Elaboration article discusses each checklist item and gives methodological background and published examples of transparent reporting. The STROBE checklist is best used in conjunction with this article (freely available on the Websites of PLoS Medicine at http://www.plosmedicine.org/, Annals of Internal Medicine at http://www.annals.org/, and Epidemiology at http://www.epidem.com/). Information on the STROBE Initiative is available at http://www.strobe-statement.org. (DOC) [file pmed.1004873.s009.doc]

STROBE Statement—Checklist of items that should be included in reports of ***cohort studies***

|  | Item No | Recommendation |
| --- | --- | --- |
| **Title and abstract** | 1 | (*a*) Indicate the study’s design with a commonly used term in the title or the abstract  Title |
|  |  | (*b*) Provide in the abstract an informative and balanced summary of what was done and what was found  Abstract |
| Introduction | | |
| Background/rationale | 2 | Explain the scientific background and rationale for the investigation being reported  Introduction, paragraphs 1-4 |
| Objectives | 3 | State specific objectives, including any prespecified hypotheses  Introduction, paragraph 5 |
| Methods | | |
| Study design | 4 | Present key elements of study design early in the paper  Methods, study design and data sources, paragraphs 1-4 |
| Setting | 5 | Describe the setting, locations, and relevant dates, including periods of recruitment, exposure, follow-up, and data collection  Methods (study design and data sources, paragraphs 1-3) |
| Participants | 6 | (*a*) Give the eligibility criteria, and the sources and methods of selection of participants. Describe methods of follow-up  Methods (study design and data sources, paragraphs 1-3) |
|  |  | (*b*) For matched studies, give matching criteria and number of exposed and unexposed Not applicable |
| Variables | 7 | Clearly define all outcomes, exposures, predictors, potential confounders, and effect modifiers. Give diagnostic criteria, if applicable  Methods (disability status, mortality, demographic information, all paragraphs) |
| Data sources/ measurement | 8* | For each variable of interest, give sources of data and details of methods of assessment (measurement). Describe comparability of assessment methods if there is more than one group  Methods (study design and data sources, paragraph 1) |
| Bias | 9 | Describe any efforts to address potential sources of bias  Methods (statistical analysis, paragraph 4) |
| Study size | 10 | Explain how the study size was arrived at  Methods (study design and data sources, paragraphs 2-3 and Figure 1) |
| Quantitative variables | 11 | Explain how quantitative variables were handled in the analyses. If applicable, describe which groupings were chosen and why  Methods (disability status, mortality, demographic information, all paragraphs) |
| Statistical methods | 12 | (*a*) Describe all statistical methods, including those used to control for confounding  Methods (statistical analysis, all paragraphs) |
|  |  | (*b*) Describe any methods used to examine subgroups and interactions  Methods (statistical analysis, paragraph 5) |
|  |  | (*c*) Explain how missing data were addressed  Methods (study design and data sources, paragraphs 2-3 and Figure 1) for a complete case analysis |
|  |  | (*d*) If applicable, explain how loss to follow-up was addressed Not applicable |
|  |  | (*e*) Describe any sensitivity analyses  Methods (statistical analysis, paragraph 4) |
| Results | | |
| Participants | 13* | (a) Report numbers of individuals at each stage of study—eg numbers potentially eligible, examined for eligibility, confirmed eligible, included in the study, completing follow-up, and analysed  Figure 1 |
|  |  | (b) Give reasons for non-participation at each stage  Figure 1 |
|  |  | (c) Consider use of a flow diagram  Figure 1 |
| Descriptive data | 14* | (a) Give characteristics of study participants (eg demographic, clinical, social) and information on exposures and potential confounders  Figure 2 |
|  |  | (b) Indicate number of participants with missing data for each variable of interest  Figure 1 |
|  |  | (c) Summarise follow-up time (eg, average and total amount)  Results, paragraph 1 |
| Outcome data | 15* | Report numbers of outcome events or summary measures over time  Results, paragraph 1 |
| Main results | 16 | (*a*) Give unadjusted estimates and, if applicable, confounder-adjusted estimates and their precision (eg, 95% confidence interval). Make clear which confounders were adjusted for and why they were included  Table 1 |
|  |  | (*b*) Report category boundaries when continuous variables were categorized  Figures 4 and 5 |
|  |  | (*c*) If relevant, consider translating estimates of relative risk into absolute risk for a meaningful time period  All results are presented on both the relative and absolute scales. |
| Other analyses | 17 | Report other analyses done—eg analyses of subgroups and interactions, and sensitivity analyses  S4 Table and S5 Table |
| Discussion | | |
| Key results | 18 | Summarise key results with reference to study objectives  Discussion, paragraphs 1-2 |
| Limitations | 19 | Discuss limitations of the study, taking into account sources of potential bias or imprecision. Discuss both direction and magnitude of any potential bias  Discussion, paragraphs 4-5 |
| Interpretation | 20 | Give a cautious overall interpretation of results considering objectives, limitations, multiplicity of analyses, results from similar studies, and other relevant evidence  Discussion, paragraphs 6-10 |
| Generalisability | 21 | Discuss the generalisability (external validity) of the study results  Discussion, paragraph 4 |
| Other information | | |
| Funding | 22 | Give the source of funding and the role of the funders for the present study and, if applicable, for the original study on which the present article is based  Acknowledgements |

*Give information separately for exposed and unexposed groups.

**Note:** An Explanation and Elaboration article discusses each checklist item and gives methodological background and published examples of transparent reporting. The STROBE checklist is best used in conjunction with this article (freely available on the Web sites of PLoS Medicine at http://www.plosmedicine.org/, Annals of Internal Medicine at http://www.annals.org/, and Epidemiology at http://www.epidem.com/). Information on the STROBE Initiative is available at http://www.strobe-statement.org.
