# Supplementary material for: Inequalities in cancer mortality between people with and without disability: A nationwide data linkage study of 10 million adults in Australia
Source: PLoS Med. 2026 Jan 5;23(1):e1004873. doi: 10.1371/journal.pmed.1004873 (PMC12768262; doi:10.1371/journal.pmed.1004873)
Supplement: Attachment — Submitted filename: R1_Response.docx [file pmed.1004873.s012.docx]

## Response to Reviewer’s Comments

Note: Page and line numbers in this response correspond to the clean version of the manuscript.

### Reviewer #1

This is a strong paper addressing an important and under-explored question - the increased cancer mortality among people with disabilities. It uses a large and well-structured dataset in Australia, which is an almost unique resource. The authors present both absolute and relative differences in cancer mortality, which is an advancement of the topic. There is a relatively stringent definition of disability - requiring assistance or supervision with daily activities - which means that people labelled with disabilities will be severely affected and so the inequities identified may be larger than for all people with disabilities. This limitation is acknowledged.

**R1.1**	My main question is the focus on cancers due to lifestyle in the results and discussion. It seems to me that a lot of the inequalities related to screening-related cancers (breast, cervical, prostate, colorectal), which the authors could also consider further.

**Response:** We have edited our Results section to emphasise results for screening-related cancers (page 8, line 245):

“Figure 4 illustrates individual cancers contributing to the overall cancer mortality rate difference (absolute inequality) by five-year age group, including screening-related cancers. For both sexes, lung cancer was the leading contributor to the absolute inequalities in mid-aged to older individuals (35 years and older). In females, breast cancer was the second major contributor in nearly all age groups, followed by colorectal cancer. In males, colorectal cancer was the second major contributor across most age groups, with prostate cancer contributing substantially in those aged 55 years and older.”

And in Discussion (page 12, line 357):

“Given the global low uptake of cancer screening programs for people with disability, common barriers need to be addressed to improve participation, including lack of autonomy, inaccessibility, financial cost, and stigma and fear.”

References added:

- Kuper H, Andiwijaya FR, Rotenberg S, Yip JLY. Principles for Service Delivery: Best Practices for Cervical Screening for Women with Disabilities. International Journal of Women's Health 2024; 16: 679-92.
- Merten JW, Pomeranz JL, King JL, Moorhouse M, Wynn RD. Barriers to cancer screening for people with disabilities: A literature review. Disability and Health Journal 2015; 8(1): 9-16.

**R1.2**	Moreover, the discussion was relatively long and could be cut by about a quarter. In particular, the section on future research was a little repetitive.

**Response:** We have edited our Discussion section down to avoid repetition.

**R1.3**	It is a small point - but in the introduction the authors could flag that another reason for higher cancer mortality among people with disabilities is that for specific conditions that are frequently disabling can also lead to cancer - such as Down Syndrome and leukaemia.

**Response:** We thank the reviewer for this helpful suggestion. As our study does not include information on disability type, we did not incorporate the suggested point into the introduction. We have noted this as a limitation and for future research (page 10, line 294):

“More detailed information on disability was not collected, therefore, we were unable to examine cancer mortality associated with specific disabling conditions, such as leukaemia-related mortality among people with Down syndrome.”

Reference added:

- Boucher AC, Caldwell KJ, Crispino JD, Flerlage JE. Clinical and biological aspects of myeloid leukemia in Down syndrome. Leukemia 2021; 35(12): 3352-60.

**R1.4**	Is it possible for the authors to explore further the role of socio-economic differences between people with and without disabilities in cancer mortality inequities? If not, could the authors reference this limitation?

**Response:** We thank the reviewer for raising this important question. We have expanded our discussion (page 10, line 309):

“Without longitudinal data, we were also unable to examine how certain factors, such as socioeconomic disadvantage, act as causes or consequences of disability. We did not stratify analyses by these factors, as this might introduce bias. For instance, if disability contributed to socioeconomic disadvantage, which in turn led to higher mortality, adjusting for or stratifying by socioeconomic position would underestimate the inequality”

Reference added:

- van Zwieten A, Tennant PWG, Kelly-Irving M, Blyth FM, Teixeira-Pinto A, Khalatbari-Soltani S. Avoiding overadjustment bias in social epidemiology through appropriate covariate selection: a primer. J Clin Epidemiol 2022; 149: 127-36.

### Reviewer #2

"Inequalities in cancer mortality between people with and without disability: a nationwide data linkage study of 10 million adults" analyzes the potential contribution of cancer, to the mortality gap between people with and without disabilities. It was found that people with disability had higher cancer mortality as compared to people without disability, and therefore proposed that interventions targeted towards people with disability should be prioritized across the cancer control pathway.

A strength of the study is its consideration of type-specific effects for cancer, with the reporting of absolute inequality estimates also raised as a novel contribution, that is relevant in resource allocation when compared to other potential contributors (e.g. neurological conditions). Concerns include the simplified definition and self-reported status of disability, and moreover the assignment of participants' disability status based on a single timepoint.

Some further issues might be considered:

**R2.1**	In the Study Design and data sources, it is stated that data was obtained from the 2011 Australian Census of Population for respondents aged 25 to 74 years (cutoff to minimize age-related disability/mortality) that responded to the disability question, together with ABS data on death registrations from 2011-2022 for mortality. As supported by the following Disability status subsection, this appears to indicate that disability status is determined at a single timepoint (2011) for all participants. However, it appears likely that disability status may change during the course of the study, especially for older participants. It might therefore be carefully justified as to how this effect is considered, in analyzing and presenting the results. If possible, estimates might be provided for the prevalence of such changes in disability status.

**Response:** We thank the reviewer for highlighting this important point. We have amended our text to clarify (page 10, line 297):

“Disability is also a dynamic status that may change over time. In our study, disability status was measured at a single point in time (the 2011 Census), which captured participants’ baseline status but not subsequent transitions. In the 5% sample of 2011 Census records linked to the 2016 Census in the Australian Census Longitudinal Dataset, about 33% of those aged 25 to 74 years who reported a disability in 2011 no longer did so in 2016, while around 3% of those without disability in 2011 reported disability in 2016. Disability status may also have changed between and beyond the two censuses. Therefore, our results should be interpreted as reflecting inequalities according to baseline disability status, rather than sustained or fluctuating status over time. Future data improvement efforts should aim to reconstruct disability dynamics at a population scale using multiple longitudinal administrative data sources, such as routine care and disability support data. This would allow better assessment of how time-varying disability influences cancer mortality.”

Reference added:

- Australian Bureau of Statistics (ABS). Australian Census Longitudinal Dataset (ACLD). 2022. https://www.abs.gov.au/about/data-services/data-integration/integrated-data/australian-census-longitudinal-dataset-acld Accessed 10 October 2025.

**R2.2**	Related to the above, it appears likely that disability status is dynamic and correlates with (cancer) mortality. For example, patients determined to be without disability when surveyed in 2011, would appear likely to be eventually considered disabled were they to develop (terminal) cancer afterwards. It might be discussed as to how such effects are addressed in the analysis.

**Response**: As discussed in our response to R2.1, our study primarily captures inequalities according to baseline disability status. We excluded the first two years of follow-up after the Census date to reduce the possibility that individuals close to the end of life were more likely to report disability (page 6, line 172). However, our study cannot account for newly acquired or fluctuating disability over follow-up, including during cancer progression. Future improvements in disability data would enable better assessment of how time-varying disability (such as onset, duration, and dynamic patterns over time) influences cancer mortality (page 10, line 305).

**R2.3**	In the Study Design and data sources subsection, it is further stated that only persons who responded to the disability question were included in this study. It might be clarified as to the census participation rate (in particular, whether it is mandatory), the disability question response rate (and any implications of the response, e.g. eligibility for disability programs/benefits), and whether this sampling may have been biased (e.g. against persons living in more-remote regions, or disabled persons themselves, etc.)

**Response:** We have amended our text (page 5, line 130):

“The Census is compulsory and had a response rate of 96.3% in 2011.”

To address the concern that missing response may not be missing at random (e.g., more likely to be people with disability), we performed a sensitivity analysis to assess the impact of missing disability response on our inequality estimates (page 7, line 205) under two extreme missing-not-at-random scenarios: all were people with disability, and all were people without disability.

Results are as follows (page 9, line 260):

“Our sensitivity analysis, which categorised all non-respondents as people without disability, showed virtually no changes in the inequality estimates compared to the main analysis. If the non-respondents were all people with disability, the inequality estimates were slightly attenuated but remained substantial (Supplementary Table 5), noting that this is an extreme missing-not-at-random scenario.”

We discussed potential biases related to this issue (page 11, line 321):

“First, we excluded 2,102,320 (16.5% of all respondents aged 25 to 74 years) who could not be linked to the population spine therefore not linked to death data. However, this exclusion is unlikely to have altered our conclusion given its minimal impact on the age structure and age-specific disability prevalence. Second, we excluded people who did not respond to the Census disability questions (n= 132,139, 1% of all respondents aged 25 to 74 years). However, sensitivity analysis showed that absolute and relative inequalities remained substantial under extreme missing-not-at-random scenarios.”

**R2.4**	In the Mortality subsection, it is then stated that cancer and cancer type-specific mortality were determined using the underlying cause of death, according to ICD-10. It might be specifically clarified as to whether Australian reporting standards under ABS would include multiple (possible) causes of death, since this is especially material to the analysis.

**Response:** ABS do include multiple causes of death data. However, for the purpose of describing cancer mortality inequalities between two groups, we used underlying cause of death. The analysis of Australian Institute of Health and Welfare on underlying and multiple causes of death shows that deaths involving cancer typically had cancer listed as the underlying cause of death.

Reference:

- Australian Institute of Health and Welfare. Multiple causes of death in Australia: an analysis of all natural and selected chronic disease causes of death 1997-2007. Canberra: AIHW; 2012. accessed 03 October 2025. Available at: <https://www.aihw.gov.au/reports/life-expectancy-deaths/multiple-causes-of-death-in-australia-an-analysis/contents/results>

**R2.5**	In Figure 2, the presented disability statistics appear to indicate that significantly more than 50% of participants aged above 65 are disabled (as opposed to non-disabled), in 2011; however, SDAC data (<https://www.abs.gov.au/statistics/health/disability/disability-ageing-and-carers-australia-summary-findings/latest-release>) states that only about 52% of people aged 65 years and over have disability in 2022, despite a general trend towards increasing disability since 2018 (and likely 2011). This seemingly large discrepancy might be clarified.

**Response:** We would like to clarify that Figure 2 presents the age structure by disability status, not disability prevalence within each age group. Disability prevalence by age group is shown in Supplementary Table 4, where prevalences are lower than those estimated by SDAC. This difference is primarily due to the different measures of disability used. We also included an overall comparison with SDAC 2012 statistics, which are closer in time to the 2011 Census (page 10, line 313):

“The Census questions had a strong conceptual basis for identifying individuals with severe functional limitations in performing core daily activities (3.5% of our study population). Using a broader disability definition that includes less severe disabilities, the estimated prevalence was 21% for ages 25-74. This may limit the generalisability of our findings to people with less severe forms of disability. However, the disability group in our study is more likely to experience socioeconomic disadvantage thus higher avoidable inequalities in cancer mortality than people with less severe disability.”

**R2.6**	In the Statistical analysis subsection, the analysis for age-standardized mortality rates is described. However, it appears that potential covariates (e.g. weight, BMI, socioeconomic status, smoking/drinking/drug use, family history of diseases etc.) relevant to mortality are not accounted for. While the possible impact of socioeconomic disadvantage is discussed as a limitation, the lack of correction for other common covariates/confounders might be addressed. In particular, have previous studies considered these factors?

**Response:** We thank the reviewer for raising this important point. Our study has a descriptive aim rather than a causal one. We do not have longitudinal data to determine whether the suggested factors are confounders (common causes of disability and mortality), mediators (on the causal pathway), or both if time-varying. For this reason, we did not adjust for them. Socioeconomic disadvantage was used as an example in our response to R1.4 to illustrate why adjustment may bias the inequality estimates.

Some previous studies have adjusted for a wide range of factors, including those suggested. While such approaches can provide useful insights, they showed substantially attenuated or even reversed inequality estimate, potentially due to over-adjustment. Future research with longitudinal data is needed to better clarify these pathways.

References added:

- van Zwieten, A., et al. (2022). "Avoiding overadjustment bias in social epidemiology through appropriate covariate selection: a primer." Journal of Clinical Epidemiology 149: 127-136.
- Mansournia, M. A., et al. (2017). "Handling time varying confounding in observational research." BMJ 359.

**R2.7**	It might be instructive to also present all-cause mortality rates stratified by disability status (as in Supplementary Table 3).

**Response**: We thank the reviewer for this suggestion. All-cause mortality rates by disability status have been reported in our previous publication. For this reason, we did not include them in the current study. We have edited the text below to clarify (page 11, line 340):

“Our previous study …. This relative inequality translates to 368 (females) and 462 (males) additional cancer deaths per 100,000 person-years, making cancer the leading contributor to absolute all-cause mortality inequality among other causes of death.”

Reference:

Yang Y, Summers P, Aitken Z, Kavanagh A, Disney G. All-cause and cause-specific mortality inequalities between people with and without disability: a nationwide data linkage study in Australia. *Lancet Public Health* 2025; **10**(1): e11-e9.

**R2.8**	The methods used for CI estimation might be briefly described in supplementary material.

**Response**: We’ve added the following description in supplementary method:

“Bootstrap confidence intervals

Bootstrapping is a statistical technique that allows us to estimate confidence intervals without relying on strict assumptions about the underlying data distribution. This was done by repeatedly resampling the observed data with replacement to create many simulated datasets. From these, we calculated the inequality estimates across all samples and use the distribution of those results to derive a confidence interval.

Reference:

Efron, B., Tibshirani, R. The Bootstrap Method for Assessing Statistical Accuracy. Behaviormetrika 12, 1–35 (1985). https://doi.org/10.2333/bhmk.12.17_1”

**R2.9**	Minor grammatical errors (e.g. "...compare[d] to those without", Line 291) might be corrected.
**Response**: We thank the reviewer for noting this. We have corrected the grammatical error.

### Reviewer #3

Thank you for the opportunity to review this interesting paper. This paper provides a clear and concise analysis of cancer mortality among people with disability using Australian mortality data. The analysis is comprehensive and well-documented, covering a wide range of aspects, including interpretations, limitations, and a discussion of inequalities indicators. I recommend that this paper would be published.
**Response**: We appreciate the reviewer’s positive assessment and recommendation for publication.

### Reviewer #4

Thank you for the opportunity to review this national study out of Australia providing a descriptive epidemiology study of cancer mortality among people reporting disability compared to those who did not report disability. There remains an importance of continued documentation of differences in cancer outcomes for adults with disabilities globally.

Major concerns

**R4.1**      Why use standardization to account for age distribution differences rather than using regression models to directly compare cancer-specific mortality rates over time between the group who reported a disability and the group that did not? As has been pointed out in other studies, using standardization to account for differences in age structure means that you cannot tell whether differences are due to a difference in age specific rates or a difference in population structure. As a result, drawing strong, actionable inferences from the data are not possible.

**Response**: We use standardisation to compare rates between people with and without disability of the same age profile. This approach ensures that the observed differences are not due to variations of age profiles between the two groups (page 6, line 177). We also presented age-specific mortality rates to ensure that important differences in age-specific mortality rates between people with and without disability were not masked in the age-standardised rates (page 7 line 193).

We chose standardisation because it provides intuitive measures of both absolute rate differences and relative rate ratios. Regression models, such as Poisson or Cox regression, typically estimate relative differences (rate ratios or hazard ratios) rather than absolute rate differences that are directly interpretable for public health and policy.

**R4.2**     Cancer mortality is a function of both the incidence and fatality of a disease. Cancer mortality estimates or comparisons without accompanying accounts of cancer incidence are less valuable. Adding estimates of cancer incidence alongside the estimates of cancer mortality would significantly strengthen the value and impact of the included work. Otherwise, it is unclear whether differences in mortality rates between those reporting disability and those that did not is caused by a greater burden of the disease or a greater progression from or disparities in management following diagnosis. As a result, drawing strong , actionable inferences from the data are not possible.

**Reference:** We thank the reviewer for this important point. While data linking cancer diagnoses to disability are not yet available in Australia, our study represents an important first step in describing inequalities in cancer mortality between people with and without disability. By documenting these inequalities, our research fills a critical evidence gap and highlights the need for linked disability-cancer diagnosis data (page 12, line 388):

“Our study examined inequalities in cancer mortality, which reflect the total disease burden but do not distinguish between differences in cancer incidence and survival. In Australia, cancer screening, diagnosis, and treatment data are not yet linked to disability data. Future data linkage with cancer registries is needed to better understand how disability influences cancer incidence, which may be reduced through prevention, and cancer survival, which can be improved through equitable care.”

**R4.3**      Disability as defined in this study suggests that disability is not a static construct or medical diagnosis and therefore changes over time, as it uses a timeframe for determining its occurrence (>6 months) and removes folks experiencing more transient forms of disability. However, after that initial differentiation, the definition of disability used in the study assumes no change in disability status over a 15-year period. It's unclear that the measures used in the study have validity or reliability in such an extended time-period and would still be reflected at the time of a cancer-related death. It is also unclear how heterogenous this population is and therefore how to direct future implications or resources, when and to whom to prevent cancer mortality (or risk of developing cancer). As a result, drawing strong, actionable inferences is more challenging.

**Response**: We agree with the reviewer that ideally, analyses should use longitudinal data with repeated measures of disability capturing the onset, duration, disability type, and level of functioning. However, such detailed population-level disability data do not exist yet in Australia or in most other countries. While there are ongoing efforts aimed at improving the availability and quality of disability data, our study provides an important first step in documenting inequalities with existing data. In our responses to comments R2.1 and R2.2, we have included discussion about implications of assessing disability status at a single time point. Despite this limitation, it is critical to begin quantifying these inequalities to inform future data development and research priorities.

**R4.4.**      The authors previously published using the same cohort, including a comparison of cancer-related mortality (overall). It is unclear why the authors differently defined the cohort between the two studies, given the rationale provided in the study under review (to ensure disability was not the cause of mortality) and similarity of goals and purposes of the published study and the one under review. Age is a determinant of both cancer incidence and disability, and the authors aim to account for differences in the age distribution between those who report and did not report a disability by standardizing the population to the age distribution of those with a disability. Why would folks additionally need to be excluded on age?

**Response**: Our previous publication on all-cause and cause-specific mortality included people aged 0-74 years. In the current study, we restricted the cohort to ages 25-74 years. We maintained the upper age limit in both studies to ensure that old age does not become the primary reason for reporting disability in older age groups. In the current study, the lower age limit was increased, as cancer mortality is predominantly concerns in adult population. In contrast, our previous study included younger age groups because we compared mortality rates due to causes of death that are also common in younger populations (e.g., neurological conditions).

Reference:

Yang Y, Summers P, Aitken Z, Kavanagh A, Disney G. All-cause and cause-specific mortality inequalities between people with and without disability: a nationwide data linkage study in Australia. *Lancet Public Health* 2025; **10**(1): e11-e9.

**R4.5.**      The rationale for labelling and focusing on "lifestyle related cancers" inappropriately emphasizes the role of individual factors with the reported disability in cancer-related mortality and places blame or responsibility on the person for their circumstances, rather than the systems or structures in place that may result in that individual's "lifestyle" or cancer outcomes.

**Response**: We thank the reviewer for highlighting this important point. We acknowledge that the term “lifestyle-related cancers” may unintentionally place blame to individuals. Our intention was to classify cancers according to established evidence for carcinogenesis, which partly reflect modifiable risk factors. We have edited our text to avoid focusing on lifestyle as personal choices (page 12, line 361):

“Our findings showed that cancers associated with risk factors such as smoking, obesity, and alcohol consumption contribute substantially to the absolute inequalities in cancer mortality between people with and without disability. These factors are shaped by broader social, environmental, and structural determinants. Studying these modifiable contributors is important because they represent potential opportunities for interventions to reduce inequalities. Further research, including qualitative research, is also needed to assess whether public health programs are inclusive and effective for people with disability, and to guide equitable strategies for prevention and care.

Smoking-related cancers made a large contribution to the inequalities observed in our study. … Addressing these inequalities requires not only inclusive prevention strategies, but also structural changes to make healthcare systems more equitable and accessible for people with disability.”

General editorial requests

**Response**: We have checked each item and made changes where applicable.

(Note: not all will apply to your paper, but please check each item carefully)

*       We ask every co-author listed on the manuscript to fill in a contributing author statement, making sure to declare all competing interests. If any of the co-authors have not filled in the statement, we will remind them to do so when the paper is revised. If all statements are not completed in a timely fashion this could hold up the re-review process. If new competing interests are declared later in the revision process, this may also hold up the submission. Should there be a problem getting one of your co-authors to fill in a statement we will be in contact. Please do not add or remove authors without first discussing this with the handling editor. You can see our competing interests policy here: <http://journals.plos.org/plosmedicine/s/competing-interests>.

*       Please upload any figures associated with your paper as individual TIF or EPS files with 300dpi resolution at resubmission; please read our figure guidelines for more information on our requirements: <http://journals.plos.org/plosmedicine/s/figures>. While revising your submission, we strongly recommend that you use PLOS's NAAS tool (<https://ngplosjournals.pagemajik.ai/artanalysis>) to test your figure files. NAAS can convert your figure files to the TIFF file type and meet basic requirements (such as print size, resolution), or provide you with a report on issues that do not meet our requirements and that NAAS cannot fix.

After uploading your figures to PLOS's NAAS tool - <https://ngplosjournals.pagemajik.ai/artanalysis>, NAAS will process the files provided and display the results in the "Uploaded Files" section of the page as the processing is complete.
If the uploaded figures meet our requirements (or NAAS is able to fix the files to meet our requirements), the figure will be marked as "fixed" above. If NAAS is unable to fix the files, a red "failed" label will appear above.
When NAAS has confirmed that the figure files meet our requirements, please download the file via the download option, and include these NAAS processed figure files when submitting your revised manuscript.

*       Please ensure that the paper adheres to the PLOS Data Availability Policy (see <http://journals.plos.org/plosmedicine/s/data-availability)>, which requires that all data underlying the study's findings be provided in a repository or as Supporting Information. For data residing with a third party, authors are required to provide instructions with contact information (web or email address) for obtaining the data. Please note that a study author cannot be the contact person for the data. PLOS journals do not allow statements supported by "data not shown" or "unpublished results." For such statements, authors must provide supporting data or cite public sources that include it.

*       We expect all researchers with submissions to PLOS in which author-generated code underpins the findings in the manuscript to make all author-generated code available without restrictions upon publication of the work. In cases where code is central to the manuscript, we may require the code to be made available as a condition of publication. Authors are responsible for ensuring that the code is reusable and well documented. Please make any custom code available, either as part of your data deposition or as a supplementary file. Please add a sentence to your data availability statement regarding any code used in the study, e.g. "The code used in the analysis is available from Github [URL] and archived in Zenodo [DOI link]" Please review our guidelines at <https://journals.plos.org/plosmedicine/s/materials-software-and-code-sharing> and ensure that your code is shared in a way that follows best practice and facilitates reproducibility and reuse. Because Github depositions can
be readily changed or deleted, we encourage you to make a permanent DOI'd copy (e.g. in Zenodo) and provide the URL.

*       Ethics statement: Please provide details on consent.

FORMATTING - GENERAL
*       Abstract: Please structure your abstract using the PLOS Medicine headings (Background, Methods and Findings, Conclusions). Please combine the Methods and Findings sections into one section.

*       At this stage, we ask that you include a short, non-technical Author Summary of your research to make findings accessible to a wide audience that includes both scientists and non-scientists. The Author Summary should immediately follow the Abstract in your revised manuscript. This text is subject to editorial change and should be distinct from the scientific abstract. Ideally each sub-heading should contain 2-3 single sentence, concise bullet points containing the most salient points from your study. In the final bullet point of 'What Do These Findings Mean?', please include the main limitations of the study in non-technical language. Please see our author guidelines for more information: <https://journals.plos.org/plosmedicine/s/revising-your-manuscript#loc-author-summary>.

*       Please express the main results with 95% CIs as well as p values. When reporting p values please report as p<0.001 and where higher as the exact p value p=0.002, for example. Throughout, suggest reporting statistical information as follows to improve clarity for the reader "22% (95% CI [13%,28%]; p</=)". Please be sure to define all numerical values at first use.

*       Please include page numbers and line numbers in the manuscript file. Use continuous line numbers (do not restart the numbering on each page).

*       Please cite the reference numbers in square brackets. Citations should precede punctuation.

FIGURES AND TABLES
*       Please provide titles and legends for all figures and tables (including those in Supporting Information files).

*       Please define all abbreviations used in each figure/table (including those in Supporting Information files).

*       Please consider avoiding the use of red and green in order to make your figure more accessible to those with color blindness.

SUPPLEMENTARY MATERIAL
*       Please note that supplementary material will be posted as supplied by the authors. Therefore, please amend it according to the relevant comments outlined here.

*       Please cite your Supporting Information as outlined here: <https://journals.plos.org/plosmedicine/s/supporting-information>

REFERENCES
*       PLOS uses the numbered citation (citation-sequence) method and first six authors, et al.

*       Please ensure that journal name abbreviations match those found in the National Center for Biotechnology Information (NCBI) databases (<http://www.ncbi.nlm.nih.gov/nlmcatalog/journals>), and are appropriately formatted and capitalised.

*       Where website addresses are cited, please include the complete URL and specify the date of access (e.g. [accessed: 12/06/2023]).

*       Please also see <https://journals.plos.org/plosmedicine/s/submission-guidelines#loc-references> for further details on reference formatting.

STUDY TYPE-SPECIFIC REQUESTS
*       Abstract: Please include the study design, population and setting, number of participants, years during which the study took place (enrollment and follow up), length of follow up, and main outcome measures.

* Please ensure that the study is reported according to the GATHER statement (available from <https://www.equator-network.org/reporting-guidelines/gather-statement)> and include the completed checklist as Supporting Information. Please add the following statement, or similar, to the Methods: "This study is reported as per the Guidelines for Accurate and Transparent Health Estimates Reporting (GATHER) statement (S1 Checklist)." When completing the checklist, please use section and paragraph numbers, rather than page numbers.

*       For all observational studies, in the manuscript text, please indicate: (1) the specific hypotheses you intended to test, (2) the analytical methods by which you planned to test them, (3) the analyses you actually performed, and (4) when reported analyses differ from those that were planned, transparent explanations for differences that affect the reliability of the study's results. If a reported analysis was performed based on an interesting but unanticipated pattern in the data, please be clear that the analysis was data driven.

*       Please state in the Methods section whether the study had a prospective protocol or analysis plan. If a prospective analysis plan (from your funding proposal, IRB or other ethics committee submission, study protocol, or other planning document written before analyzing the data) was used in designing the study, please include the relevant document(s) with your revised manuscript as a Supporting Information file to be published alongside your study and cite it in the Methods section. A legend for this file should be included at the end of your manuscript. If no such document exists, please make sure that the Methods section transparently describes when analyses were planned, and when/why any data-driven changes to analyses took place. Changes in the analysis, including those made in response to peer review comments, should be identified as such in the Methods section of the paper, with rationale.
