# Supplementary material for: Inequalities in cancer mortality between people with and without disability: A nationwide data linkage study of 10 million adults in Australia
Source: PLoS Med. 2026 Jan 5;23(1):e1004873. doi: 10.1371/journal.pmed.1004873 (PMC12768262; doi:10.1371/journal.pmed.1004873)
Supplement: Attachment — Submitted filename: R2_Response.docx [file pmed.1004873.s013.docx]

Response to Editorial Comments

Please see our response to the requests from editors in blue.

GENERAL
* Please confirm that your title complies with to PLOS Medicine's style. Your title must be nondeclarative and not a question. It should begin with main concept if possible. "Effect of" should be used only if causality can be inferred, i.e., for an RCT. Please place the study design ("A randomized controlled trial," "A retrospective study," "A modelling study," etc.) in the subtitle (ie, after a colon).

We confirm that the title complies with PLOS Medicine’s style.

* Statistical reporting: Please revise throughout the manuscript, including tables and figures.
- Please report statistical information as follows to improve clarity for the reader ""22% (95% CI [13,28]; p</=)"".
- Please separate upper and lower bounds with commas instead of hyphens as the latter can be confused with reporting of negative values.
- Please repeat statistical definitions (HR, CI etc.) for each set of parentheses.

We have revised our manuscript, tables and figures to meet the above requirements.

* Please ensure that all abbreviations are defined at first use throughout the text (including statistical abbreviations).

We confirm that the manuscript meets the above requirement.

* Please ensure that tables and figures, including those in supplementary files, are appropriately referenced in the main text.

We confirm that the manuscript meets the above requirement.

* Please review your text for claims of novelty or primacy (e.g. 'for the first time' or ‘novel’) and remove this language.

We have removed any claims of novelty or primacy.

* Please confirm that any use of statistical terms (such as trend or significant) are supported by the data, and if not please remove them. The term trend should be used only when the test for trend has been conducted.

We confirm that the manuscript meets the above requirement.

* Please provide titles and legends for all figures and tables (including those in Supporting Information files).

We confirm that the manuscript meets the above requirement.

* Please define all acronyms used in each figure or table in its corresponding legend.

We confirm that the manuscript meets the above requirement.

* Please confirm the use of patient-centered language. Please note that patient-centered language is constructed with the use of post-modified nouns (e.g. 'patients with psoriasis’ (or similar) instead of ‘psoriasis patients’) putting the person first in the sentence structure.

We confirm that the manuscript meets the above formatting requirement.

* Please review your manuscript and edit to ensure compliance with our inclusive language requirements https://journals.plos.org/plosmedicine/s/human-subjects-research#loc-categorization
We confirm that the manuscript meets the above requirement.

* Please consider including an acknowledgment of the individuals who contributed their data.

We have added the following acknowledgement:

“We acknowledge the individuals and agencies involved in the collection of Census data used in this study.”

* Please state whether you have used any author-generated code in your analysis.
we have added the following text to our “Data sharing” statement:

“Author-generated code used in this study is available from the authors upon request.”

ABSTRACT
* Please confirm that your abstract complies with our requirements, including providing all the information relevant to this study type https://journals.plos.org/plosmedicine/s/submission-guidelines#loc-abstract 
We confirm that the manuscript meets the above requirement.

* Please quantify the main results (with 95% CIs and p values).
We have added quantification of the main results with 95% CIs. We have not included p-values because point estimates and 95% confidence intervals provide both the effect size and its precision.

* Please confirm that all numbers presented in the abstract are present and identical to numbers presented in the main manuscript text.
We confirm all numbers match the main text.

* In the abstract, please include the important dependent variables that are adjusted for in the analyses.

We have included the variable adjusted for (i.e., age).

* In the last sentence of the Abstract Methods and Findings section, please describe the main limitation(s) of the study's methodology.

We have added the following limitation:

“The main limitation of the study was that disability status was measured at a single time point.”

* We suggest changing the language from ‘cancer deaths’ to ‘cancer related deaths’. Please revise throughout.

We have changed the language to cancer related deaths in the abstract and the main text.

* We suggest reporting numerators and denominators alongside percentages (as done in the Result section of the main text).
We have added numerators and denominators alongside percentages.

* We suggest reporting the average follow-up time (9.2 years on average) alongside the person-years.

We have added the average follow-up time in the abstract.

* “We found similar-sized relative inequalities in mortality due to individual cancers in both sexes.” – between individuals with and without disability?

We have revised the text to the following for clarity:

“We found similar-sized relative inequalities between individuals with and without disability in mortality due to individual cancers in both sexes.”

AUTHOR SUMMARY
* In the author summary, in the final bullet point of 'What Do These Findings Mean?', please include the main limitations of the study in non-technical language.

We have added the following point:

“The main limitation was that disability status was recorded only on the 2011 Census day, and we could not track any changes over time.”

INTRODUCTION
* Please change the heading to ‘INTRODUCTION’.

We have changed the heading.

METHODS AND RESULTS
* We suggested that you complete the GATHER checklist and report your study accordingly, but we cannot find a response to our request in the rebuttal.
* If you do not think that GATHER is the appropriate checklist for your study, please ensure that the study is reported according to the STROBE guideline, and include the completed STROBE checklist as Supporting Information. Please add the following statement, or similar, to the Methods: ""This study is reported as per the Strengthening the Reporting of Observational Studies in Epidemiology (STROBE) guideline (S1 Checklist).""
When completing the checklist, please use section and paragraph numbers, rather than page numbers.
* Please state in the Methods section whether the study had a prospective protocol or analysis plan. If a prospective analysis plan (from your funding proposal, IRB or other ethics committee submission, study protocol, or other planning document written before analyzing the data) was used in designing the study, please include the relevant document(s) with your revised manuscript as a Supporting Information file to be published alongside your study and cite it in the Methods section. A legend for this file should be included at the end of your manuscript. If no such document exists, please make sure that the Methods section transparently describes when analyses were planned, and when/why any data-driven changes to analyses took place. Changes in the analysis, including those made in response to peer review comments, should be identified as such in the Methods section of the paper, with rationale.

We have completed a STROBE checklist as Supporting Information (S1 Checklist), and have added the following statement under Methods:

“This study is reported as per the Strengthening the Reporting of Observational Studies in Epidemiology (STROBE) guideline (S1 Checklist).”

* Figure 2: Please add 'years' as a unit on the y-axis.

We have updated Figure 2 with “years” as a unit on the y-axis

* Table 1: For the ‘Rate Ratio’, please add ‘(95% CI)’ to define the statistical meaning of the numbers in brackets.

We have added “95% CI” in Table 1.

* “The mortality rate ratios ranged from 1.53 to 2.17 for females and 1.50 to 2.02 for males across individual cancers.” – We suggest referencing S2 Table here (again).

We have added a reference to S2 Table.

* Figure 4/5: Please convert any stacked bar charts to another data representation for example a table, or other type of graph. At minimum, please provide the values behind the stacked bars in supplementary tables.

The data underlying figures 4 and 5 are provided in S3 Table. We have added this clarification to legends of these two figures.

* Figure 4/5: Please define ‘Other cancers’.

We’ve added the following text to the figure legends to clarify:

- “Other cancers” include all cancer types not individually presented in Figure 4.
- “Other cancers” include all cancer types not classified as “obesity-related”, “alcohol-related”, “smoking-related”, or “lifestyle-related” in each of the four panels in Figure 5.

* Figure 5: In the description, please define ‘Obesity-related cancers’, ‘Alcohol-related cancers’, ‘Smoking-related cancers’ and ‘Lifestyle-related cancers’.

We’ve added the following text to the figure legend to clarify:

“Obesity-related cancers, alcohol-related cancers, and smoking-related cancers are classified based on evidence evaluation done by International Agency for Research on Cancer and World Cancer Research Fund. Lifestyle-related cancers are cancers related to obesity, alcohol, and smoking. Cancers related to each lifestyle exposures are not mutually exclusive.”

Relevant information was also included in Methods.

* Please confirm that you provided the unadjusted comparisons as well as the adjusted comparisons in all relevant Tables.

We confirm that crude and age-standardised comparisons are provided.

* Please specify the variables controlled for in all relevant Tables.

We confirm that the key variable (i.e., age) is specified.

DISCUSSION
* Please remove the 'conclusions' subheading from the discussion. Please also remove any other subheadings from the discussion.
We have removed all subheadings from the discussion.

GENERAL EDITORIAL REQUESTS

1) We ask every co-author listed on the manuscript to fill in a contributing author statement. If any of the co-authors have not filled in the statement, we will remind them to do so when the paper is revised. If all statements are not completed in a timely fashion this could hold up the re-review process. Should there be a problem getting one of your co-authors to fill in a statement we will be in contact. YOU MUST NOT ADD OR REMOVE AUTHORS UNLESS YOU HAVE ALERTED THE EDITOR HANDLING THE MANUSCRIPT TO THE CHANGE AND THEY SPECIFICALLY HAVE AGREED TO IT.

We will comply with this editorial request.

2) Please ensure that the paper adheres to the PLOS Data Availability Policy (see <http://journals.plos.org/plosmedicine/s/data-availability)>, which requires that all data underlying the study's findings be provided in a repository or as Supporting Information. For data residing with a third party, authors are required to provide instructions with contact information for obtaining the data. PLOS journals do not allow statements supported by "data not shown" or "unpublished results." For such statements, authors must provide supporting data or cite public sources that include it.
We included the above information in the data sharing section.

3) Please review your reference list to ensure that it is complete and correct. If you have cited papers that have been retracted, please include the rationale for doing so in the manuscript text, or remove these references and replace them with relevant current references. Any changes to the reference list should be mentioned in the rebuttal letter that accompanies your revised manuscript.

We have reviewed our reference list to ensure it is up-to-date and correct.
